# Milk! Below

**Published:** 1901-03-16

**Authors:** 


### The Hospital

A Journal of

The Medical Sciences and Hospital Administration.

Vol. XXIX.—No. 755.] Edited by Sir Henry Burdett, K.C.B., and Solomon C. Smith, M.D., M.R.C.P. [March 16, 1901.

### "MILK! BELOW."

A popular writer of fiction, in the early fifties, wishing to present two insoluble problems to his readers, inquired how much water there was in London milk, and how much milk in London cream! The same problems, with the substitution of gelatine for milk in the second of them, have for the last year occupied the attention of a Departmental Committee of the Board of Agriculture—a committee which, in its zeal for the full discharge of the duties entrusted to it, has during that time held an infinite number of sittings and has examined no less than forty-nine witnesses, of whom fifteen were "analysts," seventeen were "dairy farmers," and twelve were "representative milk traders and distributors." The chief question referred to the committee was to determine "what deficiency in any of the normal constituents of genuine milk or cream, or what addition of extraneous matter or proportion of water in any sample of milk (including condensed milk) or cream shall, for the purposes of the Sale of Food and Drugs Acts, 1875 to 1899, raise a presumption, until tho contrary is proved, that the milk or cream is not genuine." The point thus stated is one concerning which there seems to have been no general rule among analysts, some of whom have based their inquiries upon a higher standard than others, with the inevitable consequence that the proceedings of different local authorities, and of different magistrates, have not been uniform, and that presumed offenders, who would have been fined in some districts, have escaped "without a stain upon their characters" in others. The difficulty of arriving at any decision binding upon the whole country is greatly enhanced by the fact that the quality of milk varies not only in different breeds of cows and in different individuals, but at different seasons of the year, at different periods of time after calving, to some extent in accordance with the number of hours which have elapsed since the last milking, and in a doubtful manner and degree under the influence of variations in the quantity and quality of food. There is the further difficulty that it would be manifestly impossible to place the commercial standard at the highest level attained by milk of the first quality, and that to place it below this level would be a direct inducement to the fraudulent dealer to dilute, or in the language of the trade to "tone down" his best milk to the point at which it would barely comply with the requirements of the law.

The conclusions and recommendations of the committee have been published in a Blue Book which which was issued last week, and the reasons on which they were founded are stated at considerable length. We are not quite sure whether it might not have been more discreet to withhold them, on the ground that reasons are always open to criticism, while against judgments there is practically no appeal. One recalcitrant Commissioner, indeed, gives his reasons, or what appear to him to be such, at a length about equal to that of the majority report from which he dissents, and another, who signs the majority report, appends a "reservation" of three folio pages as to certain portions of it. The whole of the Commissioners, however, were agreed in their cynical dismissal of the recommendation urged upon them by certain representatives of the "trade," to the effect that there should be no fixed standard of quality at all, and that the interests of the consumer should be comfortably accommodated between the two stools furnished for their support by the "stress of competition" and by the integrity of merchants and retailers. It appeared from the evidence of analysts that milk of good quality should contain about 12 or 13 per cent. of solids, including 3·5 or 3·6 per cent. of fat; while that of Jersey cows might contain between 14 and 15 per cent. of solids, including 5·5 per cent. of fat. It is tolerably evident, therefore, that a liquid which would be called "cream" in London would be described as "milk" by the fortunate proprietor of a Jersey herd, even if it would always pass muster as deserving of the more humble appellation. The committee, on a very careful review of the whole question, decided to recommend that regulations should be made by the Board of Agriculture to the effect that in the case of any milk (other than skimmed, separated, or condensed milk), in which the total solids do not amount to 12 per cent., or in which, together with such deficiency, the milk-fat falls below 3·25 per cent., or the non-fatty solids below 8·5 per cent., a presumption shall be raised, until the contrary is proved, that it has been mixed with separated milk or with water, or that some portion of its normal content of milk-fat has been removed. They recommend that the artificial thickening of cream by gelatine or other substance shall raise a presumption that the cream is not genuine; that 9 per cent. of solids shall be required in skimmed or separated milk, and that in condensed milk the fat must not fall below 10 per cent., nor the total solid content below 25 per cent. As with fresh milk, deficiency is to raise a presumption of improper dilution. They also recommend the adoption of "effective measures" (but without stating what measures these should be) to prevent any addition of separated or condensed milk, or other extraneous matter, for the purpose of reducing the quality of genuine milk to any limits fixed by regulation of the Board of Agriculture. We confess that we do not see by what means this manifestly desirable end could be accomplished; although it is doubtless probable that some of the more enterprising milk dealers will at least profess to supply their customers with milk better than the regulation limit, in consideration of obtaining something more, probably considerably more, than the ordinary price. Such a profession would call imperatively for the careful observance of the maxim "*Caveat emptor.*"

